# Spatial Navigation Impairments Among Intellectually High-Functioning Adults With Autism Spectrum Disorder: Exploring Relations With Theory of Mind, Episodic Memory, and Episodic Future Thinking

**DOI:** 10.1037/a0034819

**Published:** 2013-11

**Authors:** Sophie E. Lind, David M. Williams, Jacob Raber, Anna Peel, Dermot M. Bowler

**Affiliations:** 1 Department of Psychology, Durham University, Durham, United Kingdom; 2 Departments of Behavioral Neuroscience and Neurology, Oregon Health & Science University; 3 Department of Psychology, City University London, London, United Kingdom

**Keywords:** autism spectrum disorder, episodic memory, episodic future thinking, spatial navigation, theory of mind

## Abstract

Research suggests that spatial navigation relies on the same neural network as episodic memory, episodic future thinking, and theory of mind (ToM). Such findings have stimulated theories (e.g., the scene construction and self-projection hypotheses) concerning possible common underlying cognitive capacities. Consistent with such theories, autism spectrum disorder (ASD) is characterized by concurrent impairments in episodic memory, episodic future thinking, and ToM. However, it is currently unclear whether spatial navigation is also impaired. Hence, ASD provides a test case for the scene construction and self-projection theories. The study of spatial navigation in ASD also provides a test of the extreme male brain theory of ASD, which predicts intact or superior navigation (purportedly a systemizing skill) performance among individuals with ASD. Thus, the aim of the current study was to establish whether spatial navigation in ASD is impaired, intact, or superior. Twenty-seven intellectually high-functioning adults with ASD and 28 sex-, age-, and IQ-matched neurotypical comparison adults completed the memory island virtual navigation task. Tests of episodic memory, episodic future thinking, and ToM were also completed. Participants with ASD showed significantly diminished performance on the memory island task, and performance was positively related to ToM and episodic memory, but not episodic future thinking. These results suggest that (contra the extreme male brain theory) individuals with ASD have impaired survey-based navigation skills—that is, difficulties generating cognitive maps of the environment—and adds weight to the idea that scene construction/self-projection are impaired in ASD. The theoretical and clinical implications of these results are discussed.

Spatial navigation refers to the ability to find one’s way around an environment. It is a skill that is essential for everyday functioning, and one that we use all the time, whether we are following a familiar route from the living room to the kitchen to make a cup of tea, or finding the way back to the novel location of our parked car after a day out shopping. Navigation involves maintaining a sense of direction and location while moving around the environment, and can be supported by external representations, such as maps, or by internal mental representations based on sensory experience (Wolbers & Hegarty, 2010). This latter type of navigation is the focus of the current article.

Researchers have identified several alternative navigation strategies in humans, including route-based and survey-based strategies (Thorndyke & Hayes-Roth, 1982). Route-based navigation relies on gradually learned, inflexible, egocentric representations of specific sequences of landmarks, junctions, and so forth. This is the type of strategy that we typically rely on when following familiar routes, such as traveling from home to work, or from the living room to the kitchen. On the other hand, survey-based navigation relies on flexible, allocentric representations, or cognitive maps (Tolman, 1948), of the layout of the environment. This is the type of strategy that we adopt when familiar route following is not possible. For example, if one’s normal route from home to work is blocked due to road works, one has to consider the layout of the wider environment (referring to one’s cognitive map) to successfully plan and execute an alternative route. These two navigation strategies appear to have unique underlying brain bases, with route-based and survey-based strategies depending on the caudate and hippocampus, respectively (Hartley, Maguire, Spiers, & Burgess, 2003).

## Theories of the Underlying Neurocognitive Basis of Navigation

Navigation is thought to be underpinned by several cognitive abilities, including long-term memory (Spiers & Maguire, 2008). Whereas route-based navigation tends to be based on (implicit) procedural memory, survey-based navigation depends on (explicit) declarative memory (Gillner & Mallot, 1998). In addition to the widely accepted role of declarative memory, two recently developed theories make more specific and novel proposals regarding the neurocognitive underpinnings of survey-based navigation.

Hassabis, Kumaran, Vann, and Maguire (2007) have proposed that navigation depends on a more basic cognitive process, termed *scene construction*—the ability to mentally generate and maintain coherent, multimodal spatial representations, which involves binding together multiple elements of an imagined scene, including contextual details such as sounds, smells, feelings, thoughts, people, and objects. This process is said to rely on a network centered on the hippocampus. In addition to underpinning navigation, scene construction is also said to underpin episodic memory (the ability to mentally reexperience past episodes) and episodic future thinking (the ability to mentally preexperience possible future episodes).

An alternative theory has been put forward by Buckner and Carroll (2007), who propose that navigation depends on the capacity for self-projection, which they define as the ability to shift from one’s current perspective to alternative perspectives. Specifically, it is claimed that navigation “involves simulating another view or mapping the environment,” which requires shifting “from the present perspective to a simulated model of an alternative world” (p. 51). According to Buckner and Carroll, self-projection is also essential for episodic memory and episodic future thinking. In this respect, the scene construction and self-projection hypotheses are overlapping. The self-projection theory departs from the scene construction theory, however, in claiming that the ability to represent others’ mental states (an aspect of theory of mind [ToM]) requires self-projection.

The key prediction emerging from these theories is that navigation abilities should be positively correlated with episodic memory, episodic future thinking, and (according to the self-projection hypothesis only) ToM, because all of these abilities depend on the same underlying mechanism/process. Thus, observation of dissociations between these abilities would provide a significant challenge to these theories. Such a challenge may come from the case of autism spectrum disorder (ASD).

## ASD: A Possible Test Case for Theories of the Neurocognitive Underpinnings of Navigation

ASD is a developmental disorder characterized by impairments in social communication and interaction and restricted and repetitive behaviors (American Psychiatric Association, 2013). At the cognitive level, ASD is characterized by impairments in episodic memory and episodic future thinking (Lind & Bowler, 2010; Lind & Williams, 2012; Lind, Williams, Bowler, & Peel, 2013; but see Crane, Lind, & Bowler, 2013), as well as ToM (Happé, 1995). Thus, individuals with ASD exhibit impairments in two of the three abilities said to rely on scene construction and three of the four abilities said to rely on self-projection.

Thus, existing evidence is consistent with the idea that scene construction and/or self-projection are impaired in ASD. This has led us to propose that at least part of the cognitive profile in ASD may be the consequence of underlying deficits in the capacity for scene construction and/or self-projection (Lind & Williams, 2012). If our hypothesis is correct—and, more widely, if the scene construction or self-projection theories are correct—we should also expect to find impairments in survey-based navigation in ASD.

However, our prediction stands in contrast to that arising from a prominent theory of the etiology of ASD, namely, the extreme male brain theory (Baron-Cohen, 2002). According to this theory, individuals with ASD represent extreme cases of the “male brain” type. The concepts of *empathizing* and *systemizing* are central to this theory. Here, empathizing refers to “the drive to identify another person’s emotions and thoughts, and to respond to these with an appropriate emotion,” whereas systemizing refers to “the drive to analyze the variables in a system to derive the underlying rules that govern the behavior of a system” (Baron-Cohen, 2002, p. 248). It is claimed that empathizing is superior to systemizing in neurotypical female brains, whereas the reverse is true in neurotypical male brains. In support of this empathizing–systemizing theory of psychological sex differences, Baron-Cohen, Knickmeyer, and Belmonte (2005) explicitly use the example of sex differences in navigation ability. A substantial literature has shown that males of several species (including humans) outperform females on tests of spatial navigation (Dabbs, Chang, Strong, & Milun, 1998; Jones, Braithwaite, & Healy, 2003). Baron-Cohen et al. (2005) highlight that “male rats perform significantly better than females do on the radial arm [maze] and Morris water maze,” that “human males also commit fewer errors and require less time to complete a ‘virtual’ maze,” and that “such differences reflect stronger systemizing in males” (pp. 819–820). Indeed, Baron-Cohen, Richler, Bisarya, Gurunathan, and Wheelright’s (2003) self-report Systemizing Quotient, which purportedly measures systemizing, includes several items relating to spatial navigation (e.g., “I find it difficult to learn my way around a new city”). Thus, spatial navigation is seen as a prototypical example of a sexually dimorphic skill (favoring males) that requires systemizing. As such, if people with ASD have extreme male brains, and are thus *hypersystemizers*, they should show intact or superior navigation skills.

## Previous Research on Navigation in ASD

To our knowledge, only three previous studies have directly explored navigation ability in ASD. However, each suffers from limitations that restrict the conclusions that can be drawn from them.

In the first of these studies, Prior and Hoffman (1990) reported a significant impairment on a computerized corridor maze task among 12 children and adolescents with ASD. However, the ASD group in this study had a significantly lower mean performance IQ than either of the two comparison groups. Given that nonverbal intelligence is a key factor in determining navigation performance, this lack of matching makes it difficult to determine whether diminished maze task performance resulted from ASD-specific navigation impairments or merely the overall lower nonverbal intelligence of the ASD group.

In the second study, Caron, Mottron, Rainville, and Chouinard (2004) reported intact or superior navigation among 16 adolescents and adults with ASD using corridor maze tasks. However, for four of their six experiments, no means, standard deviations, or inferential statistics (e.g., *F*, *t*, *p*, or effect-size values) were reported for between-groups differences, making it is difficult to interpret the results confidently.

More recently, Edgin and Pennington (2005) reported intact virtual water maze performance among 24 children with ASD. However, this finding may be attributable to the fact that the male-to-female ratio was significantly higher in the ASD group than the comparison group. Given that males generally show an advantage on spatial navigation tasks, ASD-specific deficits in navigation may have been masked in this study, because the higher proportion of males in the ASD group artificially inflated navigation performance relative to the female-dominated comparison group.

Thus, at present, the evidence from the only study of navigation in ASD among appropriately matched groups (Caron et al., 2004) does not support the view that this ability is diminished (although incomplete reporting of data by Caron et al. makes conclusions tentative, at best). However, more general task-related limitations apply to each of the existing studies of navigation in ASD, making firm conclusions about *survey-based* navigation in this disorder difficult to draw.

## Methodological Limitations of Corridor Maze and Simple Virtual Water Maze Tasks

The studies of navigation in ASD by Prior and Hoffman (1990) and Caron et al. (2004) each used corridor maze paradigms. Although such tasks provide unambiguous measures of route-based (caudate-centered) navigation strategies, it has been argued that they cannot be used as measures of survey-based (hippocampal-centered) strategies, because they can be executed using procedurally memorized sequences of turns, or stimulus–response associations, rather than topographical knowledge of the layout of an environment (Burgess, Maguire, & O’Keefe, 2002). Hence, aside from the limitations already discussed, corridor maze studies provide minimal information about survey-based navigation and have little bearing on the scene construction and self-projection theories.

Furthermore, concerns have been expressed regarding the appropriateness of water-maze-type tasks for use in humans. For example, Burgess et al. (2002) highlight the fact that highly simplistic virtual room environments, such as the one used in Edgin and Pennington’s (2005) study, lack texture (e.g., landmarks), meaning the entire environment is visible from any given vantage point. Hence, it is possible to succeed on such tasks via “simple visual pattern matching” (p. 628) without survey knowledge. Moreover, such simple environments lack a sense of immersion. Arguably, such tasks are so far removed from real-life navigation scenarios that they give us a limited picture of navigation abilities.

## The Current Study: Hypotheses and Predictions

The current study employed the memory island navigation paradigm, which involves a realistic, large-scale, virtual island environment, where task success depends on survey-based navigation strategies. This paradigm has been used in several previous studies of adults and children (e.g., Piper, Acevedo, Craytor, Murray, & Raber, 2010; Rizk-Jackson et al., 2006). In this task, participants use a joystick to find their way to several target objects positioned in different locations on the island. During an initial visible phase, participants complete several trials in which target object locations are indicated by large, easily visible flags. This phase provides an opportunity to construct a cognitive map of the environment and to learn the locations of the target objects. Here, it is possible to succeed using a locomotor guidance strategy—an online process that does not rely on survey-based navigation or long-term memory—that allows one to travel to a visible beacon (such as a tall building or, in this case, a flag) that can be kept in constant view (Foo, Warren, Duchon, & Tarr, 2005). Hence, performance during this phase indexes participants’ baseline ability to cope with extraneous, noncentral task demands, such as comprehension of task instructions and proficiency with the joystick. This phase is immediately followed by the hidden (experimental) phase in which participants complete several trials in which flag markers are no longer used. Here, participants must rely solely on their cognitive map of the environment and their memory for the location of the target object—performance depends on survey-based navigation.

Like a traditional water maze, the island consists of four quadrants, each of which is assigned a unique target object. However, unlike in most virtual water maze tasks, it is not possible to succeed through simple visual pattern matching because the environment is richly textured, with numerous buildings, trees, and other landmarks (thus, only a restricted portion of the landscape is visible from any given viewpoint). Virtual navigation tasks, such as the memory island task, are widely considered to provide a valid index of real-world navigation skills (Maguire, Burgess, & O’Keefe, 1999). Although there are notable differences between virtual and real-world tasks (e.g., in virtual tasks, the field of view is smaller and vestibular/proprioceptive information is unavailable), the cognitive maps that people develop in each context are remarkably similar (Ruddle, Payne, & Jones, 1997).

If the scene construction or self-projection hypotheses are correct, participants with ASD should perform significantly less well than comparison participants on hidden trials but not visible trials. This would indicate specific difficulties with survey-based navigation. In contrast, if the extreme male brain theory of ASD is correct, participants with ASD should show intact or superior performance on the hidden trials, reflecting strong survey-based navigation ability. Thus, the analysis of between-group differences in memory island performance provides a key opportunity for distinguishing between these theoretical positions. However, further (and more fine-grained) opportunity to distinguish between the scene construction and self-projection theories is provided by analysis of the cognitive correlates of memory island task performance.

The data presented here were collected as part of a larger study to explore the underlying basis of (a) deficits in episodic memory and episodic future thinking in ASD, and (b) predicted deficits in navigation in ASD. The episodic memory and episodic future thinking data are reported in Lind et al. (2013), which also reports data from a ToM task. Given the strong theoretical links between navigation, episodic memory, episodic future thinking, and ToM, we were interested in exploring the relation between performance on the memory island task and performance on these three tasks. We do not repeat the basic findings (i.e., between-groups differences) from these tasks here. Instead, we report only specific analyses concerning within-group relations between navigation, episodic memory, episodic future thinking, and ToM, which have not been reported elsewhere.

If the scene construction theory is correct, we should observe significant positive correlations between navigation and episodic memory and episodic future thinking, but not between navigation and ToM. On the other hand, if the self-projection hypothesis is correct, we should expect to see significant positive correlations between navigation and episodic memory, episodic future thinking, *and* ToM.

## Method

### Participants

Twenty-seven intellectually high-functioning adults with ASD (21 men) and 28 neurotypical comparison adults (21 men) participated. Participants in the ASD group had received formal diagnoses of autistic disorder (*n* = 5) or Asperger’s disorder (*n* = 22), according to the criteria of the *Diagnostic and Statistical Manual of Mental Disorders* (4th ed.; American Psychiatric Association, 2000). All participants were free from neurological/psychiatric disorders other than ASD.

Several measures were used to assess current ASD features among participants with ASD, and ASD-like features among comparison participants. First, 19 of 27 participants with ASD completed Module 4 of the Autism Diagnostic Observation Schedule–Generic (ADOS; Lord et al., 2000). ADOS assessments were conducted by trained individuals who had achieved research-level scoring reliability. Eight participants with ASD were unwilling to complete an assessment. All participants who completed the ADOS met the ASD cutoff (≥7 points).

Second, all participants completed the Autism-Spectrum Quotient (AQ; Baron-Cohen, Wheelwright, Skinner, Martin, & Clubley, 2001). All comparison participants scored below the ASD cutoff on the AQ (<26 points; Woodbury-Smith, Robinson, Wheelwright, & Baron-Cohen, 2005). All but three participants with ASD scored above the ASD cutoff on the AQ, and each of those three scored above the cutoff on the ADOS.

Finally, a relative or long-standing friend of each participant completed the Social Responsiveness Scale, second edition (SRS-2; Constantino & Gruber, 2012). Due to the lack of an appropriate or willing informant, SRS-2 data could not be obtained for two participants with ASD and two comparison participants. All comparison participants scored below the ASD cutoff (<60 points) on the SRS-2. Only two participants with ASD missed the ASD cutoff on the SRS-2 and both scored above the cutoff on the ADOS.

Participant characteristics are presented in Table 1. The groups were matched closely for verbal and nonverbal ability (assessed by the Wechsler Abbreviated Scale of Intelligence; Wechsler, 1999), age (see Table 1 for matching statistics), and sex ratio, χ^2^(1, *N* = 55) = 0.06, *p* = .808, ϕ = .03. Ethical approval was obtained from the appropriate research ethics committees. All participants gave written, informed consent, and all were paid for participation.[Table-anchor tbl1]

### Materials and Procedure

#### Memory island navigation task

**Figure 1 fig1:**
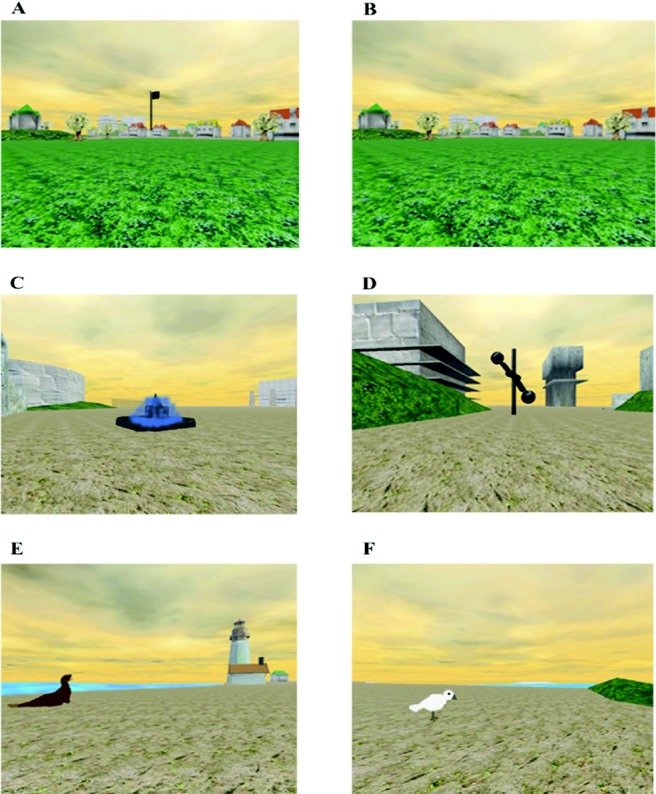
Screenshots from the memory island navigation task (A and B show the same view as it appeared in the visible and hidden conditions of the experiment, respectively; C, D, E, and F show the target objects used during the visible trials; D shows the target object used for the hidden trials).

Participants were asked to navigate within a computer-simulated, three-dimensional, island environment, measuring 347 × 287 m (see Figure 1 for screenshots). The task was presented on an RM desktop personal computer with a 22-in. LG TFT monitor, and included not only a visual (full color) depiction of the surroundings, including buildings and other landmarks, but also accompanying nature sounds (presented via Creative 265 external speakers), such as birdsong and moving water. Participants sat at a comfortable distance from the screen (∼50 cm).[Fig-anchor fig1]

Participants could explore the virtual environment using a Microsoft Sidewinder joystick, which allowed them to determine their direction and speed of travel. At the outset of the task, participants were presented with the following on-screen instructions (in addition to verbal instructions from the experimenter):

You will cruise on a virtual island. In each trial, you will start in the same position, but you may be looking in a different direction. Your mission is to find a mysterious object hidden somewhere on the island. To do that you need to look closely at what’s on the island. Try to make a map in your head of the island and where the mysterious objects are located on the island. If you cannot find the mysterious object within two minutes, an arrow will help guide you to it. Once you have found it, you must stand next to it and wait for the game to end.

##### Visible condition

During the first phase of the task, participants completed four *visible* trials. In this condition, target items were marked by large flags, which could be clearly seen from a considerable distance. Across visible Trials 1, 2, 3, and 4, the target objects were a sculpture, seal, seagull, and fountain, respectively (corresponding to D, E, F, and C, respectively in Figure 1). Each target was assigned a unique location. On a given trial, only one target was visible. At the outset of each trial, participants were instructed to locate the flag and move toward it to find the target object. If they were unable to locate it within 2 min, an arrow appeared to direct them to it. Once the object was found, the participant was required to stand next to it, and the word “Remember” appeared on the screen, prompting them to try to memorize its location.

##### Hidden condition

The visible phase was immediately followed by the hidden phase, comprising four *hidden* trials. In this condition, no flag markers were present, and participants were required to find their way to the same target object (sculpture) on each trial (the location was identical to the visible trial). The repeated use of the same target across hidden trials provided participants with continued opportunity for learning (protecting against floor effects). The target object was indicated to the participant, in the form of an on-screen visual image, at the outset of each trial. As for visible trials, if a participant was unable to locate the target object within 2 min, an arrow appeared to direct them to it.

For both visible and hidden trials, the starting location was always the center of the island. The starting orientation was varied across trials, but these variations remained constant across participants. In other words, participants started each trial facing a different direction—a feature of the task, designed to eliminate route-based strategies. Target locations were kept constant for all participants. The time taken to complete the task varied according to how quickly participants completed each trial (10 to 15 min for most participants). The experimenter remained present throughout.

##### Dependent measures

Participants’ movements were recorded in time-stamped coordinate files. Several dependent variables were calculated for each trial, including

1 Proportion of time spent within the target quadrant. This is the most commonly used performance measure on water-maze-type navigation tasks. If a participant has successfully learned the location of the target object, he or she should spend more time searching in the correct quadrant. Hence, higher scores indicate better performance and more efficient routes.
2 Latency to reach the target (s). This simply provides a measure of how long it takes a participant to reach the target object.
3 Proportion of successful trials. This provides the most basic indicator of task performance. Successful trials were defined as trials in which the target was located within 2 min (i.e., before the arrow appeared on screen to direct the participant to the target object).
4 Velocity (virtual units/s). This is not a measure of task success itself, but several other dependent variables depend, to some extent, on velocity. These variables include proportion of successful trials (it is more difficult to reach the target within 2 min if one is traveling slowly) and latency (if one is traveling quickly, one should be able to reach the target object in a shorter time). By contrast, proportion of time spent in the target quadrant is immune to the confounding effect of velocity. Hence, it is the purest measure of task success.
5 Path length (virtual units). In general, shorter paths indicate more efficient navigation—if an individual knows where they are going, they can travel directly there. However, it is important to note that, although this measure is independent of velocity, path length is not the optimal performance measure because shorter paths do not necessarily indicate task success (i.e., locating target within 2 min).

Data were not analyzed on a trial-by-trial basis because starting orientation (which varied across trials) influences the level of difficulty and would have confounded the results. Thus, for the purpose of data analysis, performance was collapsed across the four visible and four hidden trials, respectively. Average scores, rather than total scores, for each condition were used throughout (to aid comparisons with previous studies).

In the current article, we report results from each of the dependent measures described, in keeping with previous studies involving the memory island paradigm. However, the *key* measure of navigation ability is proportion of time spent in the target quadrant on hidden trials. Indeed, this is arguably the optimal measure of survey-based navigation from this task among people with ASD. As stated, proportion of time spent in the target quadrant is the only performance measure that is independent of velocity. This is particularly relevant when considering performance among individuals with ASD, as ASD is characterized by lower processing efficiency (Williams, Boucher, Lind, & Jarrold, 2013) and slower performance across a diverse range of cognitive tasks (Bowler, 1997; Sachse et al., 2013; Schmitz, Daly, & Murphy, 2007). Thus, it is highly likely that individuals with ASD will travel at lower velocity than comparison participants in the memory island task, and consequently show longer latencies and a lower proportion of successful trials. However, this would reflect generally reduced processing efficiency, rather than specific problems with spatial navigation. By focusing on proportion of time spent in the target quadrant, we are providing the most stringent and conservative test of survey-based navigation ability in ASD.

#### Episodic memory/episodic future thinking task

The episodic memory and future thinking task was based on Hassabis et al. (2007). Participants were presented with cue cards (e.g., “How you spent your last birthday,” “Something you will be doing this weekend”) and asked to remember several past personal experiences (assessing episodic memory) and imagine several future personal experiences (assessing episodic future thinking), and describe these to the experimenter. For each description, a composite experiential index score was calculated (range = 0 to 60). This provides a measure of how rich and detailed each description was. These scores were averaged across the past and future conditions, respectively, with higher scores indicating better episodic memory or future thinking. For full details of the method, see Lind et al. (2013).

#### ToM task

ToM was assessed using the animations task (Abell, Happé, & Frith, 2000), which requires participants to describe interactions between two triangles, as portrayed in a series of silent video clips. Four of the clips were intended to evoke mentalistic descriptions involving the attribution of mental states, such as belief, intention, and deception (*mentalizing* or ToM condition), and four were intended to evoke physical descriptions, involving the attribution of physical agency (*physical* or control condition). Descriptions were assigned scores of 0, 1, or 2 according to their level of accuracy. Therefore, within each condition, the maximum score was 8 points. For full details of the method see Lind et al. (2013). The animations task was employed because, unlike other classic measures of ToM (e.g., false belief tasks), it is sensitive to ToM impairments among intellectually high-functioning individuals with ASD, and variation in ToM skills among neurotypical individuals (e.g., Castelli, Frith, Happé, & Frith, 2002; Williams et al., 2013).

### Data Analysis

A standard alpha level of .05 was used to determine statistical significance. All reported significance values are for two-tailed tests (except those associated with correlation analyses, in which directional hypotheses were made). When ANOVAs or *t* tests were used, we report *r* (≥.10 = small; ≥.30 = moderate: ≥.50 = large; Cohen, 1969) or Cohen’s *d* (≥.20 = small; ≥.50 = moderate; ≥.80 = large; Cohen, 1969) values as measures of effect size.

## Results

### Memory Island Navigation Task Performance

#### Proportion of time spent in the target quadrant

**Figure 2 fig2:**
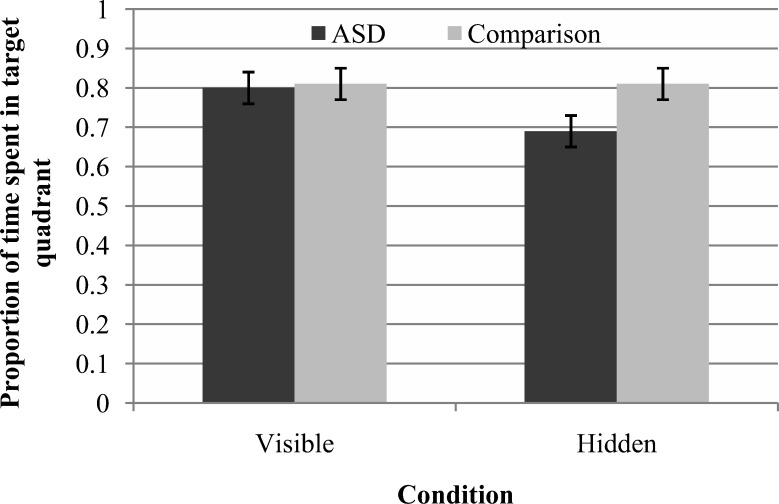
Mean proportion of time spent in the target quadrant (the primary measure of navigation ability from the memory island task) for each group in each condition (error bars represent ± 1 *SE*).

Proportion of time spent in the target quadrant was analyzed using a 2 (group: ASD, comparison) × 2 (condition: hidden, visible) mixed-design ANOVA. This revealed a nonsignificant main effect of group, *F*(1, 53) = 1.90, *p* = .174, *r* = .19, a significant main effect of condition, *F*(1, 53) = 4.71, *p* = .034, *r* = .29, reflecting poorer performance in the hidden condition, and a significant interaction between group and condition, *F*(1, 53) = 4.17, *p* = .046, *r* = .27. Figure 2 illustrates this interaction effect.[Fig-anchor fig2]

In order to establish the source of this interaction effect, follow-up *t* tests were conducted. Independent-samples *t* tests revealed that, in the visible condition, there was no significant difference between groups in proportion of time spent in the target quadrant, *t*(53) = 0.14, *p* = .887, Cohen’s *d* = 0.05 (ASD: *M* = .80, *SD* = .19; comparison: *M* = .81, *SD* = .21). However, in the hidden condition, participants with ASD spent significantly less time in the target quadrant than comparison participants, *t*(53) = 2.22, *p* = .031, Cohen’s *d* = 0.60 (ASD: *M* = .69, *SD* = .21; comparison: *M* = .81, *SD* = .19). Paired-samples *t* tests revealed that the ASD group spent significantly more time in the target quadrant in the visible condition than the hidden condition, *t*(26) = 2.54, *p* = .017, Cohen’s *d* = 0.55. However, in the comparison group, there was no significant difference in proportion of time spent in the target quadrant between the conditions, *t*(27) = 0.11, *p* = .910, Cohen’s *d* < 0.01.

#### Supplementary dependent measures

Descriptive and inferential statistics for latency, proportion of successful trials, velocity, and path length are reported in Table 2. These data were analyzed using mixed-design ANOVAs, with group (ASD, comparison) as the between-participants variable and condition (visible, hidden) as the within-participants variable. However, given that none of these supplementary dependent measures were of central *theoretical* interest, we merely summarize the results here.[Table-anchor tbl2]

These analyses revealed significant main effects of condition on all measures, reflecting overall longer latency, lower proportion of successful trials, higher velocity, and longer paths on hidden trials than visible trials. There were significant and marginally significant main effects of group on latency, proportion of successful trials, and velocity, reflecting diminished performance among ASD participants. Crucially, there were no significant interaction effects on these supplementary measures, underscoring our arguments regarding a general diminution of processing efficiency and speed in ASD: Participants with ASD showed a longer latency, lower velocity, and lower proportion of successful trials in *both* conditions. Although there was no significant main effect of group on path length, the interaction effect was marginally significant. This may reflect the fact that the difference in path length between participants with and without ASD was negligible in the visible condition (*d* = 0.15) but moderate in the hidden condition (*d* = 0.58)—participants with ASD took substantially longer paths in this condition.

### Correlations Between Navigation and Episodic Memory, Episodic Future Thinking, and ToM

Correlation analyses were used to explore the relation between navigation task performance and performance on (a) the episodic memory task, (b) the episodic future thinking task, and (c) the mentalizing (ToM) condition of the animations task. For these analyses, the navigation measure used was proportion of time spent in the target quadrant, given that this is the optimal measure of this ability.

In the first set of analyses, we explored the simple bivariate correlations between proportion of time spent in the target quadrant in the hidden condition and performance on the episodic memory/future thinking task. Among the whole sample of participants, the relation between navigation and episodic memory was positive (but small) and significant, *r* = .26, *p* = .032. Within each group separately, the association remained, but no longer reached significance (ASD: *r* = .24, *p* = .124; comparison: *r* = .17, *p* = .197).

The pattern of results concerning the relation between navigation and episodic future thinking was very similar: Again, it was positive (but small) and approached significance, *r* = .21, *p* = .065. Within each group separately, the association remained but no longer reached significance (ASD: *r* = .23, *p* = .129; comparison: *r* = .07, *p* = .361).

In the next set of analyses, we explored the bivariate correlation between proportion of time spent in the target quadrant on the hidden condition of the memory island task and scores from the mentalizing condition of animations task. This revealed a strong, positive correlation within both groups analyzed together, *r* = .66, *p* < .001, and within the ASD, *r* = .62, *p* < .001, and comparison groups, *r* = .48, *p* = .005, alone. A Fisher’s *z* test indicated that the difference between the coefficients produced by the two groups was nonsignificant, *z* = 0.70, *p* = .483.

In addition to the straightforward bivariate association between navigation and ToM, we were also interested in the possible role of any confounding factors in driving these correlations. Thus, we conducted a set of partial correlation analyses to explore the relation between (a) proportion of time spent in the target quadrant on the hidden condition, and (b) scores from the mentalizing condition of animations task, controlling for (c) proportion of time spent in the target quadrant on the *visible* condition of the memory island task, and (d) scores from the *physical* condition of animations task. This allowed us to filter out theoretically irrelevant sources of shared variance (i.e., the effect of extraneous task factors) from the analysis. Hence, this approach to analyzing the data provides a relatively uncontaminated test of the relation between ToM ability and navigation ability, independent of general (non-survey-based navigation) requirements of the memory island task *and* independent of general (non-ToM) demands inherent in the animations task. Among the groups combined, the correlation remained positive and moderately strong, *r*_ab.c.d_ = .40, *p* = .002. When analyzed in each group separately, the correlation remained significant among participants with ASD, *r*_ab.c.d_ = .51, *p* = .006, but not among comparison participants, *r*_ab.c.d_ = .27, *p* = .092. Nonetheless, the between-groups difference in the size of the correlation was nonsignificant, *Z* = 0.99, *p* = .322.

## Discussion

The aim of the current study was to assess spatial navigation ability among intellectually high-functioning adults with ASD. Arguably, the study has several advantages over the few existing studies of this ability in ASD. First, it involved the largest sample of participants with ASD (*n* = 27) of any study of spatial navigation in ASD to date, increasing confidence in the reliability of the results. Second, participant groups in the current study were matched very closely for sex, age, verbal IQ, and performance IQ, ensuring that between-groups differences in experimental (navigation) task performance are not merely the product of differences between the groups in sex, age, or intellectual ability. Finally, and perhaps most importantly, this study employed the only paradigm (the memory island task) to date that necessitates a survey-based (cognitive-map-based) navigation strategy.

In line with the primary hypothesis of the study, participants with ASD spent a significantly smaller proportion of their time in the target quadrant in the hidden condition of the memory island task, indicating impairment in spatial navigation among this group. In this respect, the difference between the groups was associated with a moderate effect size (*d* = 0.60). Crucially, despite this moderate impairment among participants with ASD in the hidden condition of the memory island task, there was no sign of any reliable difference between the groups in performance on the visible condition of the task. In this respect, the difference between the groups was associated with a negligible effect size (*d* = 0.05). Thus, the diminished performance of participants with ASD in the hidden condition was not merely due to difficulties with noncentral task demands. Rather, the problem was with survey-based navigation, specifically. Participants with ASD also showed a trend toward taking longer, less efficient paths when navigating in the hidden condition only (also associated with a moderate effect size, d = 0.58)—that is, they had to cover more ground to locate the target. In other words, participants with ASD were not only searching in the wrong areas of the island but also traveling further.

The current results contrast with those of Caron et al. (2004) and Edgin and Pennington (2005), who reported intact or superior navigation in ASD. Aside from differences in participant matching procedures, this discrepancy is probably due to the fact that (despite having impaired survey-based skills) individuals with ASD have intact route-based navigation and visual pattern matching skills, which are sufficient for success on the corridor and simple virtual water mazes employed, respectively, in these studies.

Several limitations of the current study should be considered when interpreting the results. First, although the sample size is reasonable by the standards of most ASD research, it is nevertheless relatively small by the standards of psychology more generally. Second, the sample consisted exclusively of intellectually high-functioning adults. Thus, we do not know how younger or less cognitively able participants with ASD would perform on the task. Finally, the current task employed a virtual environment. Although navigation performance among typical individuals is comparable across real-world and virtual settings, we do not know if this is also true for individuals with ASD.

The current findings have implications for theory development and for clinical practice. In terms of theory development, the current results support the notion that navigation, episodic memory, episodic future thinking, and ToM share a common underlying basis among neurotypical individuals. The fact that we observe deficits in all four of these abilities among individuals with ASD is consistent with this idea, and adds weight to the proposal that hippocampal functioning is atypical in ASD (Bowler, Gaigg, & Lind, 2011). Although dissociations between these abilities could still potentially be observed in other disorders, the finding that impairments across these domains cohere in ASD is notable. Candidates for the shared underlying basis of such impairments include diminished scene construction and/or diminished self-projection. However, because both of these theories predict impairments in navigation among people with ASD, the between-groups differences in experimental task performance observed in the current study do not allow us to distinguish between these theories. However, exploration of the cognitive correlates of navigation ability has the potential to do just that.

Both the scene construction and self-projection theories predict that navigation performance should be positively associated with episodic memory and episodic future thinking. However, only the self-projection theory implies that navigation ability should be positively associated with ToM. The finding in the current study that performance in the hidden condition of the memory island task was strongly and significantly positively associated with performance on the mentalizing condition of the animations task among both ASD and comparison participants supports the self-projection theory specifically. It is notable that performance on the memory island task was associated positively and significantly with performance on the episodic memory task only when both groups were collapsed, and not when each group was analyzed individually. However, the correlation among each group individually was highly similar in magnitude to the correlation among both groups collapsed. Therefore, the lack of statistical significance within each group individually is likely to be a product merely of limited statistical power. However, matters concerning the relation between navigation and episodic future thinking were less clear. Although, the correlation approached significance among both groups collapsed, it was modest in magnitude and only negligible among comparison participants alone. The relation between episodic memory and episodic future thinking is well established, but they are not synonymous (see Lind & Bowler, 2010). Thus, it is quite possible that navigation and episodic memory share particular cognitive underpinnings, but these are distinct from the cognitive underpinnings that are common to episodic memory and episodic future thinking.

The current results have further theoretical implications, in that they provide a challenge to the extreme male brain theory of ASD. This theory predicts that people with ASD should manifest undiminished and even superior ability on tasks (requiring systemizing) that males consistently outperform females on. Spatial navigation is perhaps the prototypical example of such a skill, with overwhelming evidence of male superiority in this ability (Dabbs et al., 1998; Jones et al., 2003). Thus, if ASD is an example of (indeed, caused by) an extreme male brain, then people with this disorder should perform at least as well as, if not better than, neurotypical comparison individuals on navigation tasks. The fact that, instead, they performed significantly less well than closely matched neurotypical participants in the current study echoes findings from recent studies that cast doubt on the validity of the extreme male brain theory. For example, Pellicano et al. (2011) explored large-scale search skills—which arguably rely on several putative systemizing abilities, including navigation-related abilities—within a laboratory-based “foraging room.” They found that children with ASD were less systematic and less efficient at foraging than typically developing comparison children. These findings could be at least partially explained by difficulties with navigation, and stand in clear contrast to the predictions of the extreme male brain theory.

The current results, in themselves, do not imply that the extreme male brain theory should necessarily be abandoned. They do, however, suggest that it, or the broader empathizing–systemizing theory of psychological sex differences among neurotypical individuals, may need some revision. If the empathizing-systemizing theory is to be preserved as an explanation for psychological sex differences, the idea that individuals with ASD have across-the-board systemizing impairments and extreme male brains in every respect needs to be rethought. Alternatively, if the extreme male brain theory is to be preserved as an explanation of ASD, the central notion of systemizing may need to be reconsidered. Specifically, navigation could no longer be classed as a systemizing skill. It would follow that the Systemizing Quotient (Baron-Cohen et al., 2003) would also require revision, to exclude the items that relate to survey-based navigation (Items 31 [“I find it difficult to learn my way around a new city”] and 49 [“I can easily visualize how the motorways in my region link up”]).

In terms of the implications of the current results for our understanding of the ASD phenotype, our observations fit with frequent anecdotal reports that individuals with this disorder often insist on sticking to well-known, familiar routes. This is typically viewed as a manifestation of the behavioral inflexibility that is diagnostic of the disorder. Therefore, our results provide an important potential explanation for this aspect of inflexible behavior, namely, that individuals with ASD have difficulties with generating cognitive maps and rely more heavily than neurotypical individuals on route-based navigation skills. Hence, taking a new route—even to a familiar location—may leave some individuals with ASD feeling lost and uncertain of their location. This could explain the high degree of anxiety experienced by some individuals with ASD when required to deviate from familiar routes. Therefore, a key clinical implication of the current findings is that people with ASD may benefit from using external representations, such as maps, to compensate for their difficulties with generating cognitive maps of the environment. In addition to encouraging the use of external representations to support navigation, it may be possible to foster the ability to generate cognitive maps among individuals with this disorder. There is evidence that purposeful training of such strategies in virtual environments can transfer into real-world benefits in navigation ability (Witmer, Sadowski, & Finkelstein, 2002). Given that survey-based navigation is diminished in ASD, but not absent, it may (and, indeed, should) be possible to foster existing capacity among individuals with this disorder. The fact that spatial navigation is so critical for day-to-day independent living suggests the potential benefits of such interventions for those individuals with ASD who experience difficulties with navigation in their daily lives may be quite significant.

We have argued that deficits in scene construction and/or self-projection in ASD may plausibly contribute to difficulties with survey-based navigation—that is, we are suggesting that there are impairments in the representational processes needed to generate cognitive maps. However, it is important to consider whether navigation difficulties may, alternatively (or additionally), stem from lack of relevant sensory input. In other words, the direction of causality may be the opposite of that assumed in the preceding paragraph: Behavioral inflexibility (specifically, sticking to well-known routes) may contribute to navigation impairments, as opposed to navigation impairments contributing to behavioral inflexibility. Effective navigation relies on appropriate sensory input in addition to the necessary representational apparatus. If a pervasive “insistence on sameness” means that a child with ASD invariably follows the same routes, they will only ever experience a limited portion of their environments, restricting their opportunities for learning about topographical layouts. Such attenuated sensory input would inevitably result in impoverished cognitive maps of real-world environments. Although it is not known whether the representational processes that underpin cognitive maps can be influenced by navigational experiences, it seems plausible to suppose that inadequate sensory input could have adverse developmental consequences on them—insufficient input may limit opportunities to practice generating richly detailed cognitive maps. In relation to the current study, the results cannot be explained in terms of inadequate *online* sensory input (because starting orientation was varied across trials, participants were prevented from repeatedly following the same route), but this does not preclude the possibility that insufficient sensory input in *development* has a persistent impact on navigation into adulthood. Clearly, further research will be required to elucidate these issues, but if this analysis turns out to be correct, it highlights the importance of intervening early in development.

## Figures and Tables

**Table 1 tbl1:** Participant Characteristics (Means, Standard Deviations, and Inferential Statistics for Between-Group Differences)

Characteristic	ASD (*n* = 27)	Comparison (*n* = 28)	*t*	*p*	*d*
Age (years)	34.64 (12.97)	33.02 (16.40)	0.41	.687	0.11
VIQ	109.78 (15.23)	112.71 (12.21)	0.79	.433	0.21
PIQ	110.89 (16.33)	113.89 (12.56)	0.61	.546	0.21
FSIQ	111.33 (15.95)	113.89 (11.17)	0.69	.492	0.19
AQ	34.15 (8.61)	12.11 (5.03)	11.54	<.001	3.12
SRS-2	96.96 (31.32)	18.77 (19.36)	10.67	<.001	3.00
ADOS-G	11.11 (2.81)				
*Note*. ASD = autism spectrum disorder; VIQ = verbal IQ; PIQ = performance IQ; FSIQ = full scale IQ; AQ = Autism Spectrum Quotient; SRS-2 = Social Responsiveness Scale, 2nd Edition; ADOS-G = Autism Diagnostic Observation Schedule-Generic.					

**Table 2 tbl2:** Descriptive (Means and Standard Deviations) and Inferential (Group [ASD, Comparison] × Condition [Visible, Hidden] Mixed-Design ANOVA) Statistics for Supplementary Memory Island Dependent Measures

Dependent measure	Condition	Descriptive statistics			Inferential statistics								
		ASD	Comparison	Total	Group			Condition			Group × Condition		
					*F*(1,53)	*p*	*r*	*F*(1,53)	*p*	*r*	*F*(1,53)	*p*	*r*
Latency to reach target (s)	Visible	88.01 (39.13)	68.85 (28.51)	78.25 (35.17)									
	Hidden	105.40 (45.80)	77.11 (29.18)	91.00 (40.49)									
	Total	96.71 (43.09)	72.98 (28.88)	84.63 (38.29)									
					7.28	.009	.35	9.00	.004	.38	1.14	.291	.15
Proportion of successful trials	Visible	.84 (.22)	.91 (.25)	.88 (.24)									
	Hidden	.72 (.24)	.86 (.21)	.79 (.23)									
	Total	.78 (.24)	.88 (.23)	.83 (.24)									
					3.78	.057	.26	6.60	.013	.33	.97	.328	.13
Velocity (virtual units/s)	Visible	7.16 (1.00)	7.88 (0.68)	7.53 (0.92)									
	Hidden	7.94 (0.67)	8.48 (0.52)	8.22 (0.65)									
	Total	7.55 (0.93)	8.18 (0.67)	7.87 (0.86)									
					12.46	.001	.44	66.89	<.001	.75	1.07	.306	.14
Path length (virtual units)	Visible	580.35 (280.62)	539.48 (257.57)	559.55 (267.42)									
	Hidden	808.62 (342.59)	640.65 (221.61)	723.11 (297.07)									
	Total	694.49 (330.88)	590.07 (243.48)	641.33 (293.08)									
					2.53	.118	.21	20.38	<.001	.53	3.03	.087	.23
*Note*. ASD = autism spectrum disorder.													

## References

[c1] Abell F., Happé F., & Frith U. (2000). Do triangles play tricks? Attribution of mental states to animated shapes in normal and abnormal development. Cognitive Development, 15, 1–16 doi:10.1016/S0885-2014(00)00014-9

[c2] American Psychiatric Association (2000). Diagnostic and statistical manual of mental disorders (4th ed., text rev.). Washington, DC: Author

[c3] American Psychiatric Association (2013). Diagnostic and statistical manual of mental disorders (5th ed.). Washington, DC: Author

[c4] Baron-Cohen S. (2002). The extreme male brain theory of autism. Trends in Cognitive Sciences, 6, 248–254 doi:10.1016/S1364-6613(02)01904-6 12039606

[c5] Baron-Cohen S., Knickmeyer R. C., & Belmonte M. K. (2005). Sex differences in the brain: Implications for explaining autism. Science, 310, 819–823 doi:10.1126/science.1115455 16272115

[c6] Baron-Cohen S., Richler J., Bisarya D., Gurunathan N., & Wheelwright S. (2003). The Systemizing Quotient: An investigation of adults with Asperger syndrome or high–functioning autism, and normal sex differences. Philosophical Transactions of the Royal Society of London: Series B: Biological Sciences, 358, 361–374 doi:10.1098/rstb.2002.1206 PMC1693117 12639333

[c7] Baron-Cohen S., Wheelwright S., Skinner R., Martin J., & Clubley E. (2001). The Autism-Spectrum Quotient (AQ): Evidence from Asperger syndrome/high-functioning autism, males and females, scientists and mathematicians. Journal of Autism and Developmental Disorders, 31, 5–17 doi:10.1023/A:1005653411471 11439754

[c8] Bowler D. M. (1997). Reaction times for mental-state and non-mental-state questions in false belief tasks by high-functioning individuals with autism. European Child & Adolescent Psychiatry, 6, 160–165 9383650 10.1007/BF00538988

[c9] Bowler D. M., Gaigg S., & Lind S. (2011). Memory in autism: Binding, self, and brain In Roth I. & Rezaie P. (Eds.), Researching the autism spectrum: Contemporary perspectives (pp. 316–346). Cambridge, UK: Cambridge University Press doi:10.1017/CBO9780511973918.013

[c10] Buckner R. L., & Carroll D. C. (2007). Self-projection and the brain. Trends in Cognitive Sciences, 11, 49–57 doi:10.1016/j.tics.2006.11.004 17188554

[c11] Burgess N., Maguire E. A., & O’Keefe J. (2002). The human hippocampus and spatial and episodic memory. Neuron, 35, 625–641 doi:10.1016/S0896-6273(02)00830-9 12194864

[c12] Caron M.-J., Mottron L., Rainville C., & Chouinard S. (2004). Do high functioning persons with autism present superior spatial abilities? Neuropsychologia, 42, 467–481 doi:10.1016/j.neuropsychologia.2003.08.015 14728920

[c13] Castelli F., Frith C. D., Happé F., & Frith U. (2002). Autism, Asperger syndrome, and brain mechanisms for the attribution of mental states to animated shapes. Brain: A Journal of Neurology, 125, 1839–1849 doi:10.1093/brain/awf189 12135974

[c14] Cohen J. (1969). Statistical power analysis for the behavioural sciences. New York, NY: Academic Press

[c16] Constantino J. M., & Gruber C. P. (2012). Social Responsiveness Scale, second edition. Torrance, CA: Western Psychological Services

[c17] Crane L., Lind S. E., & Bowler D. M. (2013). Remembering the past and imagining the future in autism spectrum disorder. Memory, 21, 157–166 doi:10.1080/09658211.2012.712976 22901078

[c18] Dabbs J. M., Chang E. L., Strong R. A., & Milun R. (1998). Spatial ability, navigation strategy, and geographic knowledge among men and women. Evolution and Human Behavior, 19, 89–98 doi:10.1016/S1090-5138(97)00107-4

[c19] Edgin J. O., & Pennington B. F. (2005). Spatial cognition in autism spectrum disorder: Superior, impaired, or just intact? Journal of Autism and Developmental Disorders, 35, 729–745 doi:10.1007/s10803-005-0020-y 16328713

[c20] Foo P., Warren W. H., Duchon A., & Tarr M. J. (2005). Do humans integrate routes into a cognitive map? Map- versus landmark-based navigation of novel shortcuts. Journal of Experimental Psychology: Learning, Memory, and Cognition, 31, 195–215 doi:10.1037/0278-7393.31.2.195 15755239

[c21] Gillner S., & Mallot H. A. (1998). Navigation and acquisition of spatial knowledge in a virtual maze. Journal of Cognitive Neuroscience, 10, 445–463 doi:10.1162/089892998562861 9712675

[c22] Happé F. G. E. (1995). The role of age and verbal ability in the theory of mind task performance of subjects with autism. Child Development, 66, 843–855 doi:10.2307/1131954 7789204

[c23] Hartley T., Maguire E. A., Spiers H. J., & Burgess N. (2003). The well-worn route and the path less travelled: Distinct neural bases of route following and wayfinding in humans. Neuron, 37, 877–888 doi:10.1016/S0896-6273(03)00095-3 12628177

[c24] Hassabis D., Kumaran D., Vann S. D., & Maguire E. A. (2007). Patients with hippocampal amnesia cannot imagine new experiences. PNAS Proceedings of the National Academy of Sciences of the United States of America, 104, 1726–1731 doi:10.1073/pnas.0610561104 PMC1773058 17229836

[c25] Jones C. M., Braithwaite V. A., & Healy S. D. (2003). The evolution of sex differences in spatial ability. Behavioral Neuroscience, 117, 403–411 doi:10.1037/0735-7044.117.3.403 12802870

[c26] Lind S. E., & Bowler D. M. (2010). Episodic memory and episodic future thinking in adults with autism. Journal of Abnormal Psychology, 119, 896–905 doi:10.1037/a0020631 20853917

[c27] Lind S. E., & Williams D. M. (2012). The association between past and future oriented thinking: Evidence from autism spectrum disorder. Learning and Motivation, 43, 231–240 doi:10.1016/j.lmot.2012.05.004

[c28] Lind S. E., Williams D. M., Bowler D. M., & Peel A. (2013). Episodic memory and episodic future thinking impairments in high-functioning autism spectrum disorder: An underlying difficulty with scene construction or self-projection? Neuropsychology. Advance online publication doi:10.1037/neu0000005 PMC3906795 24015827

[c29] Lord C., Risi S., Lambrecht L., Cook E. H., Leventhal B. L., DiLavore P. C., . . . Rutter M. (2000). The Autism Diagnostic Observation Schedule-Generic: A standard measure of social and communication deficits associated with the spectrum of autism. Journal of Autism and Developmental Disorders, 30, 205–223 doi:10.1023/A:1005592401947 11055457

[c30] Maguire E. A., Burgess N., & O’Keefe J. (1999). Human spatial navigation: Cognitive maps, sexual dimorphism, and neural substrates. Current Opinion in Neurobiology, 9, 171–177 doi:10.1016/S0959-4388(99)80023-3 10322179

[c32] Pellicano E., Smith A. D., Cristino F., Hood B. M., Briscoe J., & Gilchrist I. D. (2011). Children with autism are neither systematic nor optimal foragers. PNAS Proceedings of the National Academy of Sciences of the United States of America, 108, 421–426 doi:10.1073/pnas.1014076108 PMC3017188 21173235

[c33] Piper B. J., Acevedo S. F., Craytor M. J., Murray P., & Raber J. (2010). The use and validation of the spatial navigation Memory Island test in primary school children. Behavioural Brain Research, 209, 257–262 20219554 10.1016/j.bbr.2010.02.040 PMC2874075

[c34] Prior M., & Hoffman W. (1990). Neuropsychological testing of autistic children through an exploration with frontal lobe tests. Journal of Autism and Developmental Disorders, 20, 581–590 doi:10.1007/BF02216063 2279976

[c35] Rizk-Jackson A. M., Acevedo S. F., Inman D., Howieson D., Benice T. S., & Raber J. (2006). Effects of sex on object recognition and spatial navigation in humans. Behavioural Brain Research, 173, 181–190 doi:10.1016/j.bbr.2006.06.029 16887201

[c36] Ruddle R. A., Payne S. J., & Jones D. M. (1997). Navigating buildings in “desk-top” virtual environments: Experimental investigations using extended navigational experience. Journal of Experimental Psychology: Applied, 3, 143–159

[c37] Sachse M., Sclhlitt S., Hainz D., Ciaramidaro A., Schirman S., Walter F. P., . . . Freitag C. M. (2013). Executive and visuomotor function in adolescents and adults with autism spectrum disorder. Journal of Autism and Developmental Disorders, 43, 1222–1235 doi:10.1007/s10803-012-1668-8 23011252

[c38] Schmitz N., Daly E., & Murphy D. (2007). Frontal anatomy and reaction time in autism. Neuroscience Letters, 412, 12–17 doi:10.1016/j.neulet.2006.07.077 17196745

[c39] Spiers H. J., & Maguire E. A. (2008). The dynamic nature of cognition during wayfinding. Journal of Environmental Psychology, 28, 232–249 doi:10.1016/j.jenvp.2008.02.006 19325934 PMC2660842

[c40] Thorndyke P. W., & Hayes-Roth B. (1982). Differences in spatial knowledge acquired from maps and navigation. Cognitive Psychology, 14, 560–589 doi:10.1016/0010-0285(82)90019-6 7140211

[c41] Tolman E. C. (1948). Cognitive maps in rats and men. Psychological Review, 55, 189–208 doi:10.1037/h0061626 18870876

[c42] Wechsler D. (1999). Wechsler Abbreviated Scale of Intelligence. New York, NY: Psychological Corporation

[c43] Williams D., Boucher J. M., Lind S. E., & Jarrold C. (2013). Time-based and event-based prospective memory in autism spectrum disorder: The roles of executive function, theory of mind, and time estimation. Journal of Autism and Developmental Disorders, 43, 1555–1567 doi:10.1007/s10803-012-1703-9 23179340

[c44] Witmer B. G., Sadowski W. J., & Finkelstein N. M. (2002). VE-based training strategies for acquiring survey knowledge. Presence, 11, 1–18 doi:10.1162/105474602317343622

[c45] Wolbers T., & Hegarty M. (2010). What determines our navigational abilities? Trends in Cognitive Sciences, 14, 138–146 doi:10.1016/j.tics.2010.01.001 20138795

[c46] Woodbury-Smith M. R., Robinson J., Wheelwright S., & Baron-Cohen S. (2005). Screening adults for Asperger syndrome using the AQ: A preliminary study of its diagnostic validity in clinical practice. Journal of Autism and Developmental Disorders, 35, 331–335 doi:10.1007/s10803-005-3300-7 16119474

